# JNK1 Induces Notch1 Expression to Regulate Genes Governing Photoreceptor Production

**DOI:** 10.3390/cells8090970

**Published:** 2019-08-24

**Authors:** Mingyu Pan, Haiyang Hu, Rui Wang, Yi Zhou, Lele Zhang, Chen Wang, Quanyi Wang

**Affiliations:** 1 State Key Laboratory of Natural Medicines, School of Life Science and Technology, China Pharmaceutical University, 639 Longmian Avenue, Jiangning District, Nanjing 211198, China; 2 State Key Laboratory of Cell Biology, CAS Center for Excellence in Molecular Cell Science, Shanghai Institute of Biochemistry and Cell Biology, Chinese Academy of Sciences, University of Chinese Academy of Sciences, Shanghai 200031, China

**Keywords:** JNK1, c-Jun, Notch1, opsin, photoreceptor, retina, vision

## Abstract

c-Jun N-terminal kinases (JNKs) regulate cell proliferation and differentiation via phosphorylating such transcription factors as c-Jun. The function of JNKs in retinogenesis remains to be elucidated. Here, we report that knocking out *Jnk1*, but not *Jnk2*, increased the number of photoreceptors, thus enhancing the electroretinogram (ERG) responses. Intriguingly, Notch1, a well-established negative regulator of photoreceptor genesis, was significantly attenuated in *Jnk1* knockout (KO) mice compared to wild-type mice. Mechanistically, light specifically activated JNK1 to phosphorylate c-Jun, which in turn induced Notch1 transcription. The identified JNK1–c-Jun–Notch1 axis strongly inhibited photoreceptor-related transcriptional factor expression and ultimately impaired photoreceptor opsin expression. Our study uncovered an essential function of JNK1 in retinogenesis, revealing JNK1 as a potential candidate for targeting ophthalmic diseases.

## 1. Introduction

The retina is a thin sheet of neural tissue in the eye that senses light and transmits relevant information to the brain via the optic nerve. The vertebrate retina consists of six types of neurons and one type of glial cells (Müller glial cells), which form three cellular layers: photoreceptors in the outer nuclear layer (ONL); horizontal, bipolar, and amacrine interneurons and Müller glial cells in the inner nuclear layer (INL); and ganglion and displaced amacrine cells in the ganglion cell layer (GCL). These cell types play specific roles in the process of vision [1,2]. The detection of light stimuli is mediated by photoreceptors, which contain two basic subtypes: rods and cones. Rod photoreceptors use rhodopsin to mediate night vision and can respond to single light quanta, whereas cone photoreceptors use opsins to respond to different wavelengths of light and mediate color perception [3].

Photoreceptors are generated from multipotent progenitors [4,5]. Cones are early-born cells, with production peaking at E14.5 and E15.5 in the central and peripheral areas of the retina, respectively. The rods peaks are produced centrally, at P0, and between P0 and P2 in the peripheral parts of the retina [6]. Crx [7,8,9], Otx2 [10], Rxrg [11], Neurod1 [12,13], Thrb2 [14,15], and Nrl [16,17] are all important transcriptional factors controlling photoreceptor formation and differentiation. Additionally, Notch signaling has been established as playing a pivotal role in photoreceptor genesis, which is essential for the transition from multipotent progenitor to photoreceptor precursor [18,19]. Conditional deletion of Notch1 signaling components increases the production of both rod and cone photoreceptor precursors [20,21,22]. Furthermore, the inhibition of Notch signaling induces the expression of several important transcriptional factors in most progenitors of the mouse retina, such as Otx2 and Crx [20,22,23]. Although the role of Notch signaling in photoreceptor genesis is well known, it remains to be elucidated how Notch is regulated in retinogenesis.

JNKs (c-Jun N-terminal kinases) are members of the MAP (Mitogen-activated Protein) kinase family, including JNK1, JNK2, and JNK3. JNK1 and JNK2 are ubiquitously expressed, whereas JNK3 is predominantly expressed in the brain, heart, or testes [24]. JNKs are activated through phosphorylation by the MAPK (Mitogen-activated Protein Kinase) kinases MKK4 or MKK7. Activated JNK phosphorylates a large range of substrates, including c-Jun, ATF-2 (Activating Transcription Factor 2), Elk-1, p53, and other molecules [25]. JNKs play important roles in cell proliferation, differentiation, apoptosis, stress response, and inflammation, via phosphorylating c-Jun and related molecules [26]. Previously, JNKs have been reported to affect the optic fissure in mice [27]. However, the role of JNKs in retinogenesis remains to be further explored.

In this study, we observed that both the production of photoreceptor cells and photoreceptor-mediated ERG (electroretinogram) responses were enhanced in *Jnk1* knockout (KO) mice. Mechanistically, light specifically activated JNK1 to phosphorylate c-Jun. The activated c-Jun induced Notch1 transcription, which impaired the expression of photoreceptor-related transcription factors, as well as the expression of photoreceptor opsins. The JNK1–c-Jun–Notch1 axis and cognate downstream regulatory network changes might be some of the underlying mechanisms regulating photoreceptor production.

## 2. Materials and Methods

### 2.1. Mice

C57BL/6 mice were purchased from the Model Animal Research Center of Nanjing University. The mice were maintained under specific pathogen-free (SPF) conditions at the Center for New Drug Safety Evaluation and Research, China Pharmaceutical University. *Jnk1* KO and *Jnk2* KO mice [28,29] were kindly provided by Dr. Lijian Hui. These strains were maintained on a C57BL/6 background. Age-matched C57BL/6 mice were used as a control. All animal experiments were performed in accordance with the National Institutes of Health Guide for the Care and Use of Laboratory Animals. The protocol was approved by the Institutional Animal Care and Use Committee of China Pharmaceutical University and the Institutional Ethics Committee of China Pharmaceutical University (Approval Number: 2019-08-001).

### 2.2. Cell Culture 

The HEK293 cell line was obtained from the American Type Culture Collection (ATCC). The 661W cell line was a gift from Dr. Xin Zhang. HEK293 and 661W cell lines were maintained in Dulbecco’s Modified Eagle’s Medium (DMEM) containing 10% fetal bovine serum (FBS) under a humidified atmosphere of 5% CO_2_ at 37 °C. Cultured cells were released by trypsin and passaged every 2 days.

### 2.3. Antibodies and Reagents 

TPA and SP600125 were purchased from Beyotime Biotechnology. Papain was purchased from Sigma Aldrich (St. Louis, MO, USA). DNase I was purchased from Roche. The following antibodies were used: anti-JNK1 (sc-136205, Santa Cruz Biotechnology, Santa Cruz, CA, USA), anti-JNK2 (sc-271133, Santa Cruz Biotechnology), anti-S-opsin (ab229786, Abcam, Cambridge, UK), anti-M-opsin (NB110-74730, Novus, Centennial, CO, USA), anti-Rhodopsin (NB120-3267, Novus), anti-Notch1 (D6F11, Cell Signaling), anti-neurofilament (ab223343, Abcam), anti-c-Jun (60A8, Cell Signaling), anti-c-Jun (sc-74753, Santa Cruz Biotechnology), anti-p-c-Jun ser63 (54B3, Cell Signaling), anti-p-c-Jun ser73 (D47G9, Cell Signaling), anti-β-actin (A5316, Sigma Aldrich), normal mouse IgG (sc-2025, Santa Cruz Biotechnology), anti-JNK (sc-7345, Santa Cruz Biotechnology), and anti-p-JNK (81E11, Cell Signaling).

### 2.4. Real-Time PCR 

Total cellular RNA was isolated using TRIzol (Invitrogen, Carlsbad, CA, USA) according to the manufacturer’s instructions. The quantification of gene transcripts was performed by real-time PCR using SYBR Green PCR mix (Applied Biosystems). All values were normalized to the level of *β-actin* mRNA. The primers used are listed below:

*β-actin*: sense (AAAGACCTGTACGCCAACAC),
antisense (GTCATACTCCTGCTTGCTGAT);
*c-Jun*: sense (GCAAGCCCTGAAGGAAGAG),
antisense (GTCAT ACTCCTGCTTGCTGAT);
*Neurod1*: sense (GACGGGGTCCCAAAAAGAAAA),
antisense (GCCAAGCGCAGTGTCTCTATT);
*Crx*: sense (GTTCAAGAATCGTAGGGCGAA),
antisense (TGAGATGCCCAAAGGATCTGT);
*Rxrg*: sense (CATGAGCCCTTCAGTAGCCTT),
antisense (CGGAGAGCCAAGAGCATTGAG);
*Notch1*: sense (CCGTGTAAGAATGCTGGAACG),
antisense (AGCGACAGATGTATGAAGACTCA);
*Opn1sw*: sense (CAGCCTTCATGGGATTTGTCT),
antisense (CAAAGAGGAAGTATCCGTGACAG);
*Opn1mw*: sense (ATGGCCCAAAGGCTTACAGG),
antisense (AAGGGACCTTTGGTGCTGTT);
*Notch2*: sense (GAGAAAAACCGCTGTCAGAATGG),
antisense (GGTGGAGTATTGGCAGTCCTC);
*Notch3*: sense (TGCCAGAGTTCAGTGGTGG),
antisense (CACAGGCAAATCGGCCATC);
*Wnt2b*: sense (CCGACGTGTCCCCATCTTC),
antisense (GCCCCTATGTACCACCAGGA);
*Wnt5a*: sense (CAACTGGCAGGACTTTCTCAA),
antisense (CATCTCCGATGCCGGAACT);
*Rho*: sense (CCCTTCTCCAACGTCACAGG),
antisense (TGAGGAAGTTGATGGGGAAGC);
*Nrl*: sense (TCCCAGTCCCTTGGCTATGG),
antisense (CACCGAGCTGTATGGTGTG);
*Onecut2*: sense (GGCTTCCGTCCATGAACAAC),
antisense (CGAAATTGGGGCTGAGCATTTT);
*Chx10*: sense (CTGAGCAAGCCCAAATCCGA),
antisense (CGCAGCTAACAAATGCCCAG);
*Stx1a*: sense (AGAGATCCGGGGCTTTATTGA),
antisense (AATGCTCTTTAGCTTGGAGCG);
*Vim*: sense (CGGCTGCGAGAGAAATTGC),
antisense (CCACTTTCCGTTCAAGGTCAAG);
*Sox9*: sense (CGGAACAGACTCACATCTCTCC),
antisense (GCTTGCACGTCGGTTTTGG);
*Rbpms*: sense (CCCGTAGGCTTTGTCAGTTTT),
antisense (GTAAAGTGCAGGTACTGTGAGC);
*Prkca*: sense (GTTTACCCGGCCAACGACT),
antisense (GGGCGATGAATTTGTGGTCTT);

### 2.5. Western Blot 

Cell pellets were collected and resuspended in RIPA (Radio-Immunoprecipitation Assay) buffer (50 mM Tris-HCl (pH 7.4), 150 mM NaCl, 1 mM EDTA (Ethylene Diamine Tetraacetic Acid), 0.5% NP40, 0.25% Na-deoxycholate, 1 mM Na_3_VO_4_, 0.1% SDS (Sodium dodecyl sulfate), and 0.1 mM PMSF (Phenylmethanesulfonyl fluoride); Roche complete protease inhibitor set). The resuspended cell pellet was vortexed for 20 s and then incubated on ice for 20 min, followed by centrifugation at 12,000 rpm for 15 min. Afterward, supernatants were collected for subsequent western blot analysis. Retinas, which were isolated from mice, were homogenized with RIPA buffer and centrifuged, and then supernatants were collected for subsequent western blot analysis.

### 2.6. Subcellular Fractionation

Harvested 661W cells were resuspended in 200 μL of buffer A (10 mM HEPES (N-2-hydroxyethylpiperazine-N-ethane-sulphonicacid), 1.5 mM MgCl_2_, 10 mM KCl, 0.5 mM DTT (dl-1,4-Dithiothreitol), and 1 mM PMSF (PH 7.9)) containing 0.1% Nonidet P-40 and were incubated on ice for 15 min. The lysates were mixed and centrifuged immediately at 4000 rpm for 5 min to save supernatants containing the cytosolic fraction. The pellet was washed with 500 μL buffer A twice. Nuclear proteins were extracted by resuspending the nuclear pellets in 100 μL of buffer B (20 mM HEPES, 25% glycerol, 0.42 M NaCl, 1.5 mM MgCl_2_, 0.2 mM EDTA, 0.5 mM DTT, and 2 mM PMSF (PH 7.9)), holding them on ice for 0.5–2 h. They were vortexed every 10 min. The lysates were centrifuged at 12,000 rpm for 15 min to save supernatants containing the nuclear fraction. Both cytosolic and nuclear fractions were subjected to western blotting.

### 2.7. Histology and Immunohistochemistry

For histology, eyes from wild-type, *Jnk1* KO, and *Jnk2* KO mice were enucleated, fixed in buffered mixed aldehydes (3% paraformaldehyde and 2% glutaraldehyde in PBS, pH 7.4), and embedded in paraffin. Sections of 5 μm were stained with H & E. For immunohistochemistry, eyes from wild-type, *Jnk1* KO, and *Jnk2* KO mice were enucleated, fixed in buffered 4% PFA (4% paraformaldehyde, in PBS, pH 7.4), and embedded in paraffin. Eyes were cut into 5-μm sections. After dewaxing and rehydration, the sections were soaked in sodium citrate buffer for heat-induced epitope retrieval and incubated with 10% goat serum for 1 h to block the nonspecific binding sites. Then, sections were incubated with anti-S-opsin antibody (ab229786, Abcam, 1:200), anti-M-opsin antibody (NB110-74730, Novus, 1:400), and anti-Rhodopsin antibody (NB120-3267, Novus, 1:300) overnight at 4 °C, followed by incubation with HRP (Horseradish Peroxidase) secondary antibodies for 1 h. The sections were developed by using a diaminobenzidine substrate kit (TIANGEN) and counterstained with hematoxylin. Images were obtained with an Olympus BX41 microscope. 

### 2.8. Immunofluorescence

Here, 661W cells were plated on coverslips in 2-cm dishes: 24 h later, cells were treated with or without light for 1 h. Coverslips with the cells were washed once with PBS and fixed in 3.7% formaldehyde in PBS for 15 min. After permeabilization with Triton X-100 (0.25%) in PBS for 15 min, cells were blocked with PBS containing BSA (5%) for 1 h and then incubated with primary antibodies overnight at 4 °C. After three separate washes, cells were incubated with secondary antibody for 1 h and then stained with DAPI for 2 min. The coverslips were washed extensively and fixed on slides. Eyes from wild-type, *Jnk1* KO, and *Jnk2* KO mice were enucleated, fixed in buffered mixed aldehydes (3% paraformaldehyde and 2% glutaraldehyde, in PBS, pH 7.4), and embedded in paraffin. For immunofluorescence, eyes were cut into 5-μm sections. After dewaxing and rehydration, the sections were soaked in sodium citrate buffer for heat-induced epitope retrieval and incubated with 10% goat serum for 1 h to block the nonspecific binding sites. Then, sections were incubated with anti-S-opsin antibody (ab229786, Abcam, 1:200), anti-M-opsin antibody (NB110-74730, Novus, 1:200), and anti-Rhodopsin antibody (NB120-3267, Novus, 1:200) overnight at 4 °C, followed by incubation with secondary antibody for 1 h and then staining with DAPI for 2 min. Images were captured using a Nikon Inverted Microscope Ts2/Ts2R (Nikon ECLIPSE Ts2/Ts2R).

### 2.9. Chromatin Immunoprecipitation Assay

The chromatin immunoprecipitation (ChIP) assay was performed by essentially following the manufacturer’s protocol (SimpleChIP^®^ Enzymatic Chromatin IP Kit, Agarose Beads, #9002, Cell Signaling). The following antibodies were used: 1 μg anti-c-Jun (sc-74753, Santa Cruz Biotechnology) and 1 μg normal mouse IgG (sc-2025, Santa Cruz Biotechnology). The genomic region contained a strong affinity binding site: 5′-CCCCACCCTTGCCAAAAGTGGGTTGGCGGCAGCTCCAAGCGCCTTAGATCACCGAGTTACCATCGCTAGAGCAGCAACAGAAAGGACTAGGGCTGTGCCTGCAGGCCCCACCCCTGCAGAGACATTGAGCACAGTGGGAAGAACACTGCAGAGGAAGTAATGAAAAACGTGAGGCCCCTAAGGCCCTCAAGACCCTGTTATTCAATTACCATCCCCAGAGAAAGGGGAAACCCCAGGGACTGCCCATGACAGGGTGCTCCCGTAACATACAGAGAGTCACATGTGGATTCCAAACAGTTCATGAGCATCTGTAAAGGCAGGCTGGCTCCCTGCCCTAAGAGGCTCAGGGTATTGACATGGTGGGTATTAGTTGGCTATGGGTCAGGGGAGGCCCAGCATGAAGAAAATACCAAGGGAAGGCACTCTTATCAGCAGAAAGAAGGCTTGG-3′. For quantitative PCR assays, ChIP DNA was amplified using sense (5′-CCCCACCCTTGCCAAAAGTGGGTTG-3′) and antisense (5′-CCAAGCCTTCTTTCTGCTGATAAGAGTGC-3′) primers. The data were analyzed by the following formula: fold change enrichment = 2^−(*Ct IP*-*Ct IgG*)^.

### 2.10. Plasmid Constructs

The full-length pr was amplified from mouse retinal genome DNA by standard PCR with sense primer 5′-AAAAAGCTTGGTTCGGAAGAAAGACGACT-3′ and antisense primer 5′-AAACTCGAGGGGGTGGGAACGGTTTTCA-3′. The product was cloned into the luciferase reporter vector, pGL6-TA (Beyotime Biotechnology). The full-length pr, which contained 449 bp, was divided into 4 fragments (A, B, C, D): ΔA-pr was truncated of its A fragment, ΔB-pr was truncated of its B fragment, ΔC-pr was truncated of its C fragment, and ΔD-pr was truncated of its D fragment.

### 2.11. Transfection and Luciferase Assay

HEK293 cells were transfected with pGL6-TA reporter constructs and pSV-RL Renilla internal control plasmids using Lipofectamine 2000 (Invitrogen) according to the manufacturer’s instructions. After 48 h, cells were treated with 100 ng/mL TPA for 30 min or 20 μmol/mL SP600125 for 2 h, and then cells were treated with 100 ng/mL TPA for 30 min, with DMSO treated as the control. Cells were lysed and luciferase activity was assayed with a dual-luciferase system by normalization to Renilla activity (Promega, Madison, WI, USA).

### 2.12. Cell Isolation and Flow Cytometry

P30 retinas from wild-type and *Jnk1* KO mice were dissected and incubated for 1 h at room temperature in dissociation solution (0.6% papain and 0.1% DNaseI in PBS), and then digested cell suspension was washed with PBS solution and filtered with a 45-μm cell strainer to a single-cell suspension. The cells were resuspended to approximately 1–5 × 10^6^ cells/mL in ice cold PBS, which contained 5% FCS (fetal calf serum) and 1% sodium azide. Then, the primary antibodies, such as anti-S-opsin antibody (ab229786, Abcam, 1:200), anti-M-opsin antibody (NB110-74730, Novus, 1:200), and anti-Rhodopsin antibody (NB120-3267, Novus 1:200), were added and incubated for 30 min on ice in the dark. The cells were washed 3 times by centrifugation at 300× *g* for 5 min and were resuspended in ice cold PBS containing 5% BSA and 1% sodium azide. The fluorochrome-labeled secondary antibody Alexa Fluor^®^R647 (FcMACS, 1:400) was added and incubated for 30 min on ice in the dark. Then the cells were washed 3 times by centrifugation at 300× *g* for 5 min and were resuspended again in ice cold PBS, which contained 5% BSA and 1% sodium azide. Cells were analyzed with a MACSQuant Analyzer 10 (Miltenyi Biotec, Bergisch Gladbach, Germany). Flow cytometry analysis was done with FlowJo software (7.2, Becton, Dickinson & Company, Franklin Lakes, NJ, USA).

### 2.13. Electroretinogram (ERG) Recording

ERG responses were recorded in two groups, including 4 *Jnk1* KO and 4 control 1-month-old littermates and 4 *Jnk1* KO and 4 control 3-month-old littermates. All animals were recorded under the same settings and conditions. Mice were dark-adapted overnight before an ERG was performed. Under weak red light, mice were anesthetized with an intramuscular injection of ketamine (100 μg/g body weight). Pupils were dilated with tropicamide phenylephrine eye drops, and corneas were kept moist with a drop of hypromellose. During ERG recording, mice were tested under dark adaptation first. Then, they were exposed to full-field scotopic flashes of 1.3 ms duration, presented by the Espion E2 system and a Color Dome Ganzfeld stimulator (Diagnosys) at different intensities: 0.003, 0.01, 0.03, 0.1, 0.3, 1.0, 3.0, and 10 cd.s/m^2^. Flash stimuli above 10 cd.s/m^2^ were delivered by a Xenon lamp, and those below 10 cd.s/m^2^ were delivered by a green (525 nm) LED (Light Emitting Diode). Mice were light-adapted with a saturating background (green, 20 cd.s/m^2^) for 5 min, and five levels of stimuli were used for the photopic recordings (0.3, 1.0, 3.0, 10, and 30 cd.s/m^2^). The a-wave amplitude was measured from baseline to the first negative peak, and the b-wave amplitude was measured from the a-wave trough to the next positive peak.

### 2.14. Statistical Analysis

Statistical analysis was performed using GraphPad Prism 6.0 (GraphPad software, San Diego CA, USA), Image pro plus 6.0 (Media Cybernetics, Rockville, MD, USA), and Microsoft Excel computer programs. The results are expressed as mean ± SEM for experiments conducted at least in triplicate. The Wilcoxon–Mann–Whitney test was used to assess the difference between two groups, and a value of *p* < 0.05 was considered to be statistically significant.

## 3. Results

### 3.1. Knocking Out JNK1 Promoted Retinogenesis

**Figure 1:**
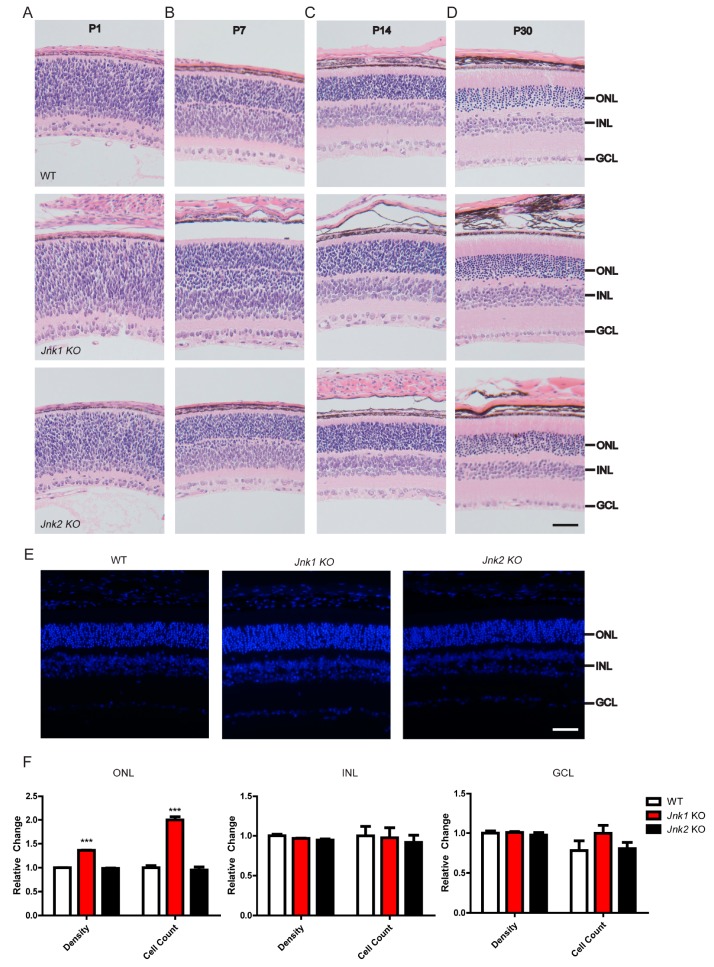
JNK1 ablation led to an increase in the outer nuclear layer (ONL). (**A**–**D**) Hematoxylin and eosin (H & E) staining of wild-type (WT), *Jnk1* knockout (KO), and *Jnk2* KO retinal sections from P1, P7, P14, and P30 mice. Scale bars: 50 μm. (**E**) WT, *Jnk1* KO, and *Jnk2* KO retinal sections from P30 mice were stained with DAPI (DNA-binding dye, 4’,6-diamidino-2-phenylindole) and imaged by immunofluorescence microscopy. Scale bars: 50 μm. (**F**) The density and cell number of the ONL, inner nuclear layer (INL), and ganglion cell layer (GCL) were quantified from equally sized fields in similar retinal regions for each retina at P30. The results are presented relative to wild-type retinal sections. Graphs show the mean ± SEM (Standard error of the mean), and the data shown are representative of three retinas. *** *p* < 0.001 (Wilcoxon–Mann–Whitney test).

To explore the effect of JNKs on retinogenesis, hematoxylin and eosin (H & E) staining of wild-type, *Jnk1* KO, and *Jnk2* KO littermates was performed at P1 (postnatal day), P7, P14, and P30, respectively (Figure 1A–D). We confirmed that JNK1 and JNK2 were indeed depleted in the *Jnk1* KO mice and *Jnk2* KO mice, respectively (Figure S1A,B). Retinas from *Jnk1* KO mice and *Jnk2* KO mice exhibited normal cell layer lamination: the outer plexiform layer, inner plexiform layer, and GCL were of similar thickness to the corresponding layers of wild-type retinas and showed no obvious morphological abnormalities. The ONL, which consists of rod and cone photoreceptors, was subtly increased in *Jnk1* KO mice. Furthermore, immunofluorescence of wild-type, *Jnk1* KO, and *Jnk2* KO littermates was carried out at P30 (Figure 1E). We determined the density and cell count of the ONL, INL, and GCL (Figure 1F). Interestingly, both the density and cell count of the ONL from *Jnk1* KO mice were significantly higher than those of the wild-type mice and *Jnk2* KO mice, whereas no apparent changes were observed in the INL or GCL layers. Retinas from *Jnk2* KO mice showed no obvious changes compared to retinas from wild-type mice. These data suggest that JNK1, not JNK2, might regulate the production of photoreceptors.

### 3.2. The Number of Photoreceptors Increased in Jnk1 KO Mice

**Figure 2:**
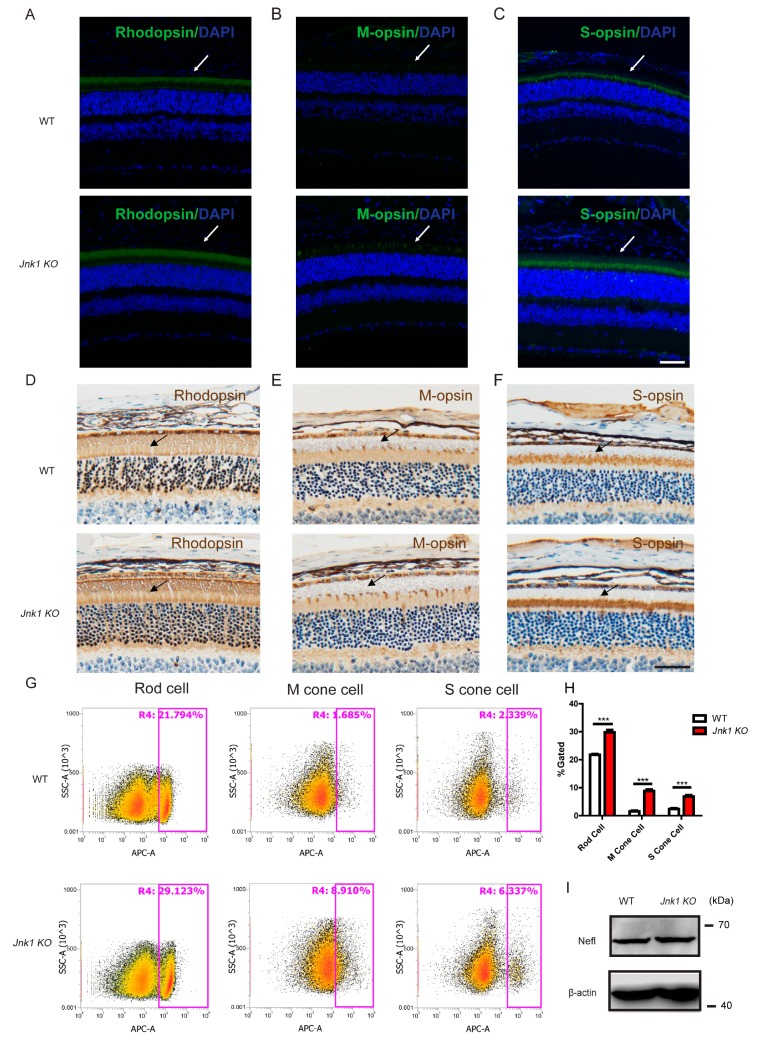
Increased production of photoreceptors in *Jnk1* KO mice. (**A**–**C**) Section immunofluorescence on WT and *Jnk1* KO retinas at P30: (A) Rhodopsin, (B) M-opsin, (C) S-opsin. Arrows point to these proteins. Scale bars: 50 μm. (**D–F**) Section immunohistochemistry on WT and *Jnk1* KO retinas at P30: (D) Rhodopsin, (E) M-opsin, (F) S-opsin. Arrows point to these proteins. Scale bars: 50 μm. (**G**,**H**) Rhodopsin-positive cells (Rod cells), S-opsin-positive cells (S cone cells), and M-opsin-positive cells (M cone cells) from WT and *Jnk1* KO mice retinas were quantified by flow cytometry. Graphs show the mean ± SEM, and the data shown are representative of three retinas. *** *p* < 0.001 (Wilcoxon–Mann–Whitney test). (**I**) Eyes were isolated from WT and *Jnk1* KO mice at P30. Tissue homogenate was collected for western blot analysis using an anti-Nefl (Neurofilament Light) antibody to indicate ganglion cells.

To confirm that JNK1 influenced the production of photoreceptors, immunofluorescence and immunohistochemistry were carried out on P30 wild-type and *Jnk1* KO retinas using photoreceptor markers (M-opsin/S-opsin for cone photoreceptor cells and Rhodopsin for rod photoreceptor cells). As expected, the expressions of the three opsins were significantly higher in *Jnk1* KO retinas than in wild-type retinas (Figure 2A–F). Western blot and real-time PCR analysis of the three markers substantiated the observations (Figure S2A,B). Consistently, the flow cytometry analysis revealed that rod photoreceptors in *Jnk1* KO retinas displayed a 1.4-fold increase compared to wild-type retinas (Figure 2G,H). Notably, there was a 2.7-fold increase of S-opsin-positive cells and a 5-fold increase of M-opsin-positive cells in *Jnk1* KO retinas compared to wild-type retinas. These data indicated that *Jnk1* modulated the production of photoreceptors.

During mouse retinogenesis, retinal ganglion cells (RGCs) are generated first, followed by cone photoreceptors and other cell types. To explore whether JNK1 alters the production of all early-born retinal cells, we used western blot and immunofluorescence to detect retinal ganglion cell marker (Nefl) expression in wild-type and *Jnk1* KO retinas at P30 and observed no difference in these retinas (Figure 2I and Figure S2C). Furthermore, real-time PCR experiments were carried out to detect the expression level of horizontal cell markers (onecut2), bipolar cell markers (Chx10 and PKCα), amacrine cell marker (Syntaxin I), Müller cell markers (Sox9 and Vimentin), and ganglion cell markers (RBPMS). We observed no expression difference in these cell-type markers between wild-type and *Jnk1* KO retinas (Figure S2D). Taken together, these data indicated that JNK1 specifically regulated the production of photoreceptors.

### 3.3. Enhanced ERG Responses in the Jnk1 KO Mice

**Figure 3:**
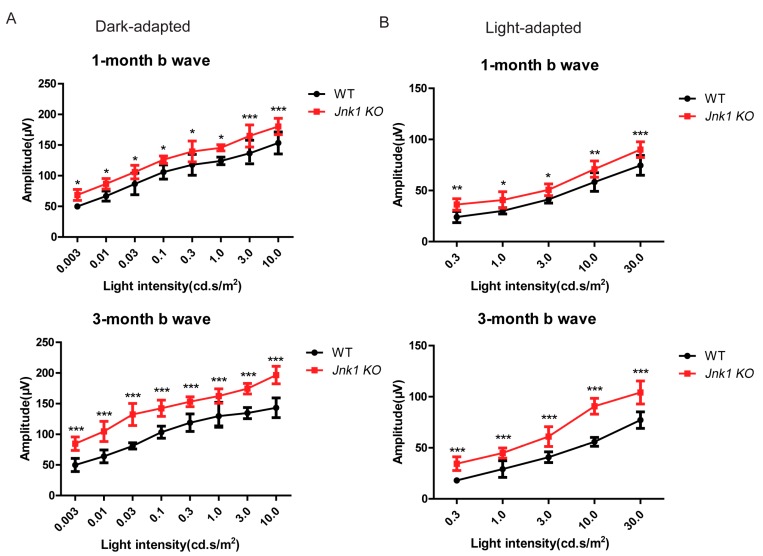
Enhanced ERG (electroretinogram) responses in the *Jnk1* KO mice. (**A**) Amplitudes of ERG b-waves elicited from WT and *Jnk1* KO mice aged 1 and 3 months. (**B**) Amplitudes of ERG b-waves elicited from WT and *Jnk1* KO mice aged 1 and 3 months. Differences between WT and *Jnk1* KO animals were significant at all flash intensities. Graphs show the mean ± SEM, and the data shown are representative of four mice. * *p* < 0.05, ** *p* < 0.01, *** *p* < 0.001 (Wilcoxon–Mann–Whitney test).

To evaluate retinal function in vivo, we recorded ERG responses under various stimulus intensities in dark- and light-adapted conditions from 1-month- and 3-month-old wild-type and *Jnk1* KO mice. At both ages, under the dark-adapted conditions, the amplitude of the ERG b-waves for *Jnk1* KO mice was significantly higher than that for wild-type mice, indicating enhanced rod-mediated activity in *Jnk1* KO mice (Figure 3A). As for light-adapted ERG responses, the results were the same, which suggested that cone-mediated activity was also enhanced (Figure 3B). These observations were consistent with the increased number of photoreceptors observed in *Jnk1* KO mice.

### 3.4. Light Activated JNK1, but Not JNK2, in 661w Cells

**Figure 4:**
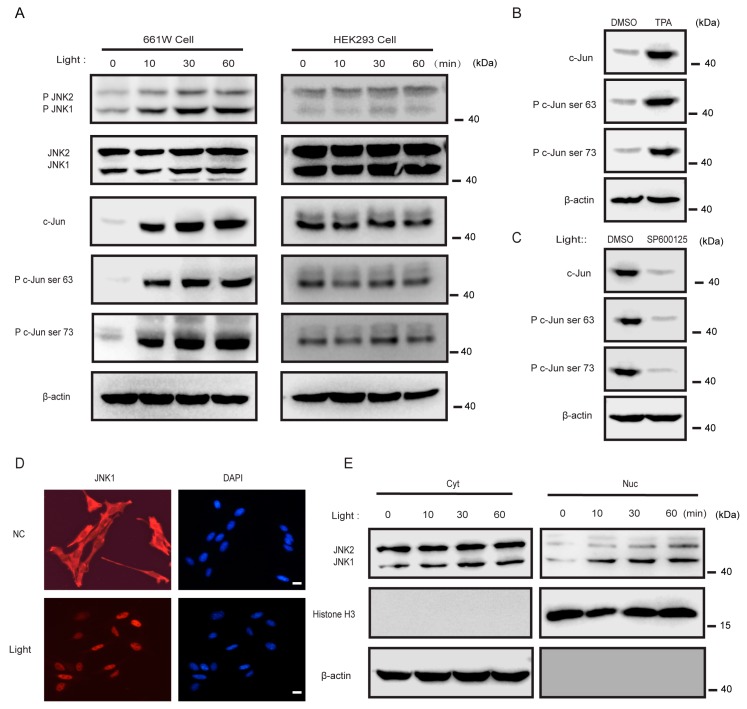
Light activated JNK1, not JNK2, in 661w cells: (**A**) 661W cells and HEK293 cells were treated by light for the indicated times. Cell lysates were collected for western blot analysis of p-JNK, JNK, c-Jun, p-c-Jun (ser63), p-c-Jun (ser73), and β-actin. (**B**) Here, 661W cells were treated with DMSO (dimethyl sulfoxide) and 100 ng/mL phorbol 12-myristate 13-acetate (TPA) for 30 min. Cell lysates were collected for western blot analysis of c-Jun, p-c-Jun (ser63), p-c-Jun (ser73), and β-actin. (**C**) Here, 661W cells were treated with DMSO and 20 μmol/mL SP600125 for 2 h and then treated by light for 30 min. Cell lysates were collected for western blot analysis of c-Jun, p-c-Jun (ser63), p-c-Jun (ser73), and β-actin. (**D**) Here, 661W cells were treated by light for 30 min and then stained with DAPI (the DNA-binding dye), an antibody specific for JNK1, and imaged by immunofluorescence microscopy. Scale bars: 10 μm. (**E**) Here, 661W cells were treated by light for 30 min. Nuclear (Nuc) and cytoplasmic (Cyt) fractions were separated and then collected for western blot analysis of JNK.

Light is indispensable for the vascular development of the eye [30]. Photoreceptors are light-sensitive cells [31,32]. By taking advantage of previously published RNA-seq (RNA-Sequencing) data (GSE106820) [33], we found that *Jra*, the orthologous gene of c-Jun in mice, was significantly induced after 3 h of blue light exposure in photoreceptors of both 1- and 6-day post-eclosion flies (Figure S3). We wondered if light could also influence the activation of JNK in photoreceptors in mice. To explore this, we used light to stimulate the photoreceptor cell line 661W (derived from murine retina photoreceptors) or control HEK293 cells and then checked the activation of JNK1. Interestingly, light stimulated the phosphorylation of JNK1 in a time-dependent manner. In addition, light triggered the phosphorylation of Ser-63 and Ser-73 on c-Jun, as expected [34,35]. However, the light did not activate JNK1 in the HEK293 cells (Figure 4A). Notably, similar effects were not observed for JNK2 in 661W cells, indicating that light may specifically activate JNK1 (Figure 4A). Phorbol 12-myristate 13-acetate (TPA) has been reported to activate JNKs [36]. As expected, TPA also stimulated the activation of JNK1 in 661W cells (Figure 4B).

Using the JNK-specific inhibitor SP600125 [37], it was observed that this inhibitor blocked the dual phosphorylation of c-Jun induced by light stimulation (Figure 4C). It is known that activated JNK translocates from the cytoplasm to phosphorylate c-Jun in the nucleus [35]. Immunofluorescence and western blot analysis revealed that light enhanced the translocation of JNK1 into the nucleus, but did not enhance the translocation of JNK2 (Figure 4D,E). These observations established that light specifically induced the activation of JNK1, but not JNK2, in 661W cells.

### 3.5. JNK1 Attenuated the Transcription of Genes Important for Photoreceptors

**Figure 5:**
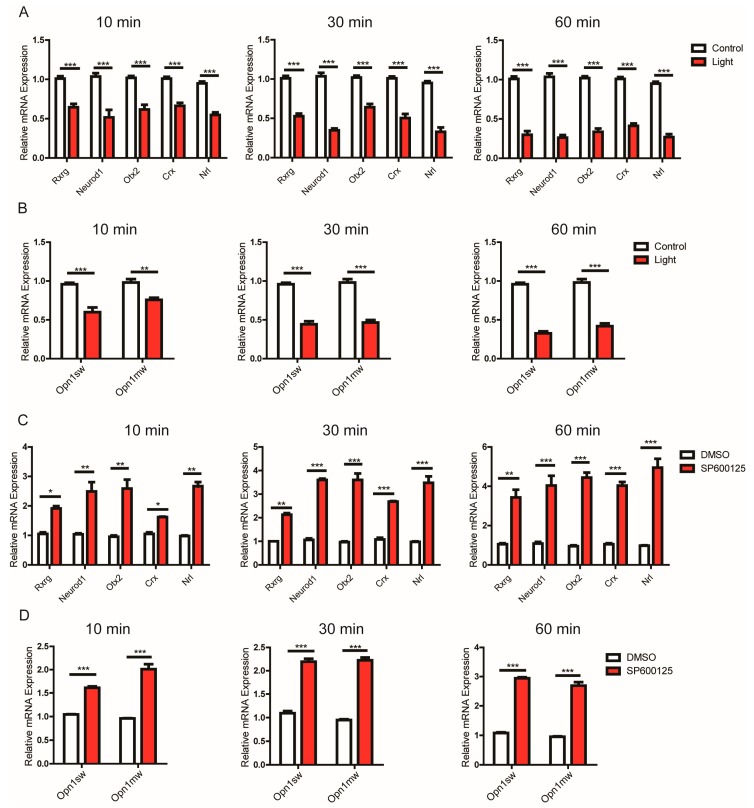
JNK1 attenuated the expression of photoreceptor-related genes in 661w cells: (**A**,**B**) 661W cells were treated by light for the indicated times, and 661W cells were treated with no light as a control. The alternation of *Rxrg*, *Neurod1*, *Otx2*, *Crx, Nrl*, *Opn1sw*, and *Opn1mw* mRNAs was then measured by quantitative PCR. (**C**,**D**) Here, 661W cells were treated with DMSO and 20 μmol/mL SP600125 for 2 h and were then treated by light for the indicated times. The alternation of *Rxrg*, *Neurod1*, *Otx2*, *Crx, Nrl*, *Opn1sw*, and *Opn1mw* mRNAs was measured by quantitative PCR. Graphs show the mean ± SEM, and the data (A–D) shown are representative of three independent experiments. * *p* < 0.05, ** *p* < 0.01, *** *p* < 0.001 (Wilcoxon–Mann–Whitney test).

We went on to explore how JNK1 impaired the photoreceptor opsin expression. Stimulating 661W cells with light significantly reduced the expression of transcription factors essential for photoreceptor fate (Figure 5A), which included Otx2, Crx, Neurod1, Rxrg, and Nrl [7,8,9,10,11,12,13,16]. Additionally, we measured *Opn1sw* (S-opsin) and *Opn1mw* (M-opsin) mRNA levels in light-stimulated 661W cells (Figure 5B). As expected, a decrease of *Opn1sw* and *Opn1mw* mRNA levels was observed. In contrast, reducing the JNK1 activation using the SP600125 inhibitor boosted the expression of the above-mentioned transcription factors, and consequently, the mRNA levels of *Opn1sw* and *Opn1mw* also increased (Figure 5C,D). These observations suggest that the JNK1 activation triggered by light suppressed the expression of the transcription factors essential for photoreceptors, which in turn attenuated the expression of the opsins.

### 3.6. The Suppressive Effect of JNK1 Was Dependent on Notch1

**Figure 6:**
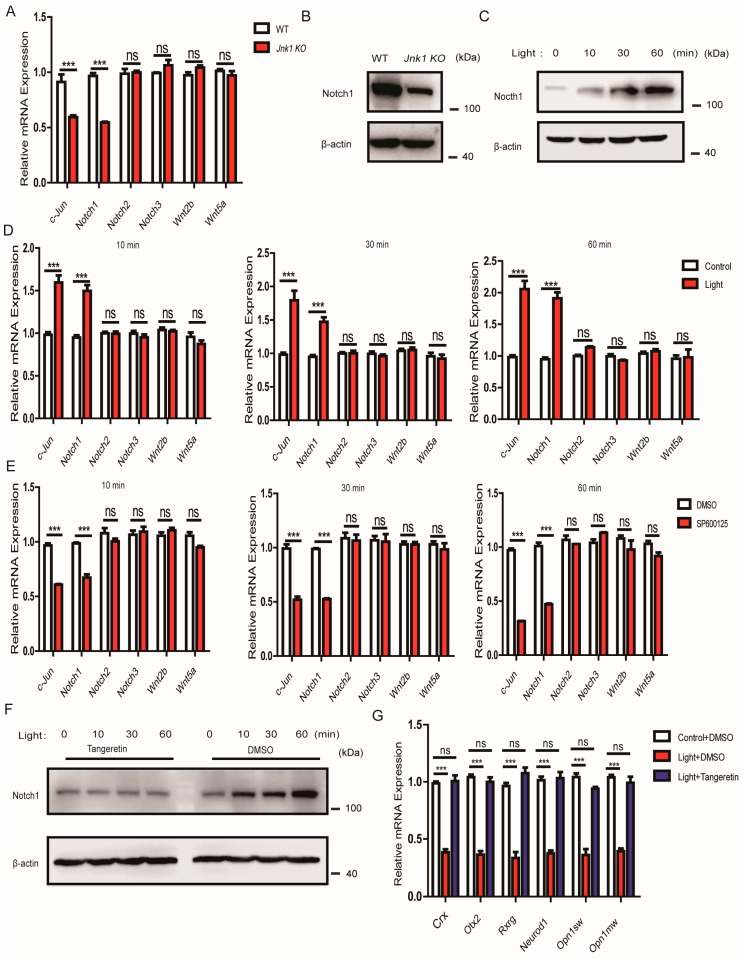
The function of JNK1 was dependent on Notch1. (**A**) Quantitative PCR analysis of *c-Jun*, *Notch1*, *Notch2*, *Notch3*, *Wnt2b*, and *Wnt5a* mRNA in WT and *Jnk1* KO retinas at P30. (**B**) Eyes were isolated from WT and *Jnk1* KO mice at P30. Tissue homogenate was collected for western blot analysis using anti-Notch1. (**C**) Here, 661W cells were treated with light for the indicated times. Cell lysates were collected for western blot analysis of Notch1 and β-actin. (**D**) Here, 661W cells were treated with light for the indicated times, and 661W cells without light stimulation were a control. Then, the alternation of *c-Jun*, *Notch1*, *Notch2*, *Notch3*, *Wnt2b*, and *Wnt5a* mRNA was measured by quantitative PCR. (**E**) Here, 661W cells were treated with DMSO and 20 μmol/mL SP600125 for 2 h and were then treated by light for the indicated times. The alternation of *c-Jun*, *Notch1*, *Notch2*, *Notch3*, *Wnt2b*, and *Wnt5a* mRNA was then measured by quantitative PCR. (**F**) Here, 661W cells were treated with DMSO and 50 μmol/mL Tangeretin for 24 h and were then treated by light for the indicated times. Cell lysates were collected for western blot analysis of Notch1 and β-actin. (**G**) 661W cells were treated with DMSO and 50 μmol/mL Tangeretin for 24 h and were then treated by light for the indicated times. The alternation of *Rxrg*, *Neurod1*, *Otx2*, *Crx*, *Opn1sw*, and *Opn1mw* mRNAs was measured by quantitative PCR. Graphs show the mean ± SEM, and the data (A,D,E,G) shown are representative of three independent experiments: ns, not significant; *** *p* < 0.001 (Wilcoxon–Mann–Whitney test).

We checked the possible alterations of the Wnt [38,39,40] and Notch [20,21,22] signaling pathways, which are important for photoreceptor fate. Notably, the mRNA levels and protein abundance of *Notch1* were significantly decreased in the *Jnk1* KO mice (Figure 6A,B). Consistently, light stimulation promoted the expression of Notch1 in 661W cells in a time-dependent manner (Figure 6C,D). In contrast, JNK-specific inhibitor SP600125 reversed the stimulatory effects on Notch1 expression (Figure 6E).

Tangeretin is an inhibitor of Notch1 gene expression [41]. Therefore, we used Tangeretin to test whether JNK1 attenuated photoreceptor-related transcription factors by influencing Notch1. It was observed that Tangeretin reversed the increase of the expression of the photoreceptor-related transcription factors, or opsins, when stimulating 661W cells with light (Figure 6F,G), indicating that JNK1 functioned upstream of Notch1.

### 3.7. JNK1 Regulated Notch1 Transcription via Activating c-Jun

**Figure 7:**
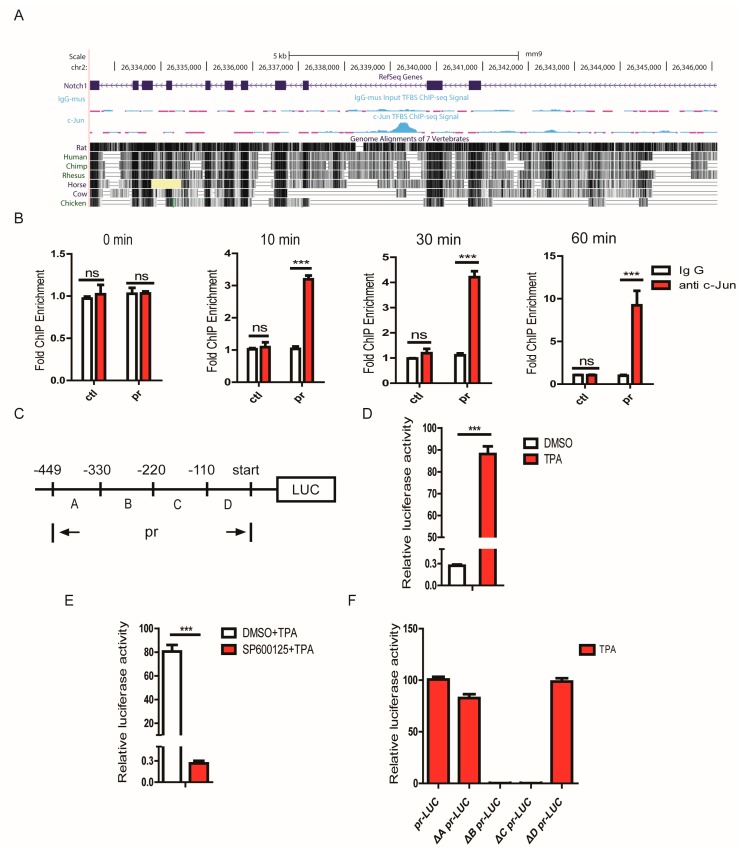
JNK1 regulated Notch1 transcription via activating c-Jun. (**A**) The chromatin immunoprecipitation (ChIP)-seq signal of c-Jun and corresponding control IgG in the region of Notch1. The four tracts show the Notch1 gene structure, IgG ChIP-seq signal, c-Jun ChIP-seq signal, and overall region sequence conservation of seven vertebrate species. (**B**) 661W cells were treated with light for the indicated times. Chromatin immunoprecipitation analysis of c-Jun DNA binding in the transcription regulatory region of Notch1. (**C**) A full-length pr was inserted into the luciferase reporter vector, pGL6-TA (The luciferase reporter vector contained minimal TA promoter). This is a schematic representation of the pr deletion constructs used in the following experiments. (**D**) Here, pr-Luc (Luciferase) reporter plasmids and pSV-RL (The luciferase reporter vector) Renilla internal control plasmids were transfected into HEK293 cells, and 48 h later, cells were treated with DMSO and 100 ng/mL of TPA for 30 min. Then, a luciferase assay was performed. (**E**) The pr-Luc reporter plasmids and pSV-RL Renilla internal control plasmids were transfected into HEK293 cells, and 48 h later, cells were treated with DMSO and 20 μmol/mL SP600125 for 2 h and then treated with DMSO and 100 μg/mL TPA for 30 min. (**F**) Here, pr-Luc or its truncation mutants were transfected into HEK293 cells, and 48 h later, cells were treated with 100 ng/mL TPA for 30 min. Then, a luciferase assay was performed. Graphs show the mean ± SEM, and the data (B,D,E,F) shown are representative of three independent experiments: ns, not significant; *** *p* < 0.001 (Wilcoxon–Mann–Whitney test).

We speculated that JNK1 regulated Notch1 expression by activating c-Jun. To explore this possibility, we first tested whether c-Jun could directly modulate Notch1 transcription. Based on previously published chromatin immunoprecipitation (ChIP)-seq data [42,43,44], we focused on pr (the 449-bp fragment, position: chr2:26339064-26339512, mouse genome mm9) of the Notch1 transcription regulatory region, which possibly harbors the binding site for c-Jun (Figure 7A). ChIP assays demonstrated that the binding of c-Jun to the transcription regulatory region of Notch1 was increased in a time-dependent manner upon light stimulation in 661W cells (Figure 7B), which indicated that JNK1 activated c-Jun to bind to the transcription regulatory region of Notch1. Furthermore, we performed a luciferase reporter assay to confirm that c-Jun directly regulated the transcription of Notch1, and a pGL6-TA vector was used to construct a recombinant plasmid carrying the firefly luciferase reporter gene under the control of the Notch1 transcription regulatory region, which was responsive to c-Jun activation (Figure 7C). Compared to the DMSO control, TPA triggered the activation of c-Jun, which markedly increased luciferase activity in HEK293 cells transfected with the recombinant plasmid (Figure 7D). In contrast, the inhibition of JNK1 activity with the JNK-specific inhibitor SP600125 suppressed a TPA-induced increase in luciferase activity (Figure 7E). To pinpoint the binding site of c-Jun in the Notch1 transcription regulatory region, we created a series of Notch1 transcription regulatory region truncations, including ΔA-pr (lack of A fragment), ΔB-pr (lack of B fragment), ΔC-pr (lack of C fragment), and ΔD-pr (lack of D fragment) (Figure 7C). Interestingly, the luciferase reporter assay indicated that the constructs lacking an A domain or D domain remained responsive to a TPA-induced increase in luciferase activity. Notably, the elimination of either the B domain or C domain resulted in a significant decrease in luciferase activity (Figure 7F). In line with this result, all three c-Jun binding sites predicted using TRANSFAC (TRANScription FACtor database) were within the B domain and C domain regions (Figure S4). Collectively, our results revealed that JNK1 regulated Notch1 expression at the transcriptional level via activating c-Jun, which bound to the transcription regulatory region of Notch1.

## 4. Discussion

**Figure 8:**
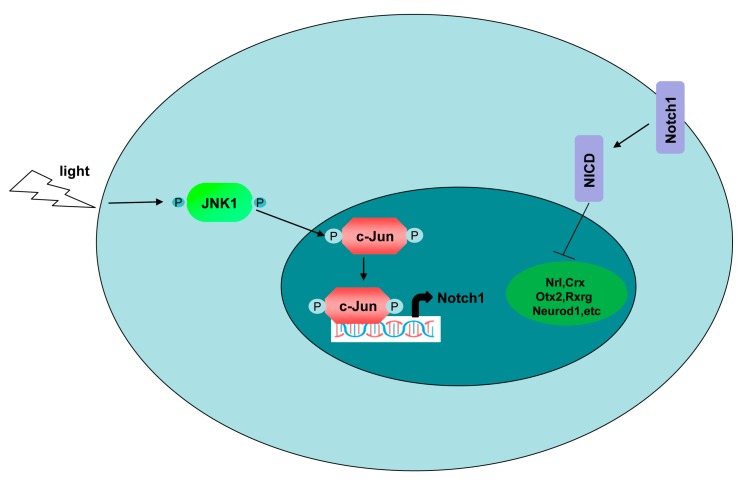
Schematic diagram of the JNK1–c-Jun–Notch1 axis. Upon Light stimulation, JNK1 was activated and translocated from the cytoplasm to phosphorylate c-Jun in the nucleus. Phosphorylated c-Jun was activated and promoted Notch1 transcription by binding to the transcriptional regulatory region of Notch1. Notch1 is a well-known negative regulator of photoreceptor genesis through inhibiting photoreceptor-related transcription factors (Nrl, Crx, Otx2, Rxrg, Neurod1, etc). Taken together, JNK1 regulated photoreceptor genesis through Notch1 via c-Jun.

JNK signaling is well established as regulating cell proliferation and differentiation. However, its potential function in modulating retinogenesis has remained largely unknown. In this study, we characterized JNK1 to specifically control the production of photoreceptor cells in retinogenesis. Unexpectedly, JNK2 apparently did not play any role in the retinogenesis. Several lines of evidence substantiated our claims. In vivo, JNK1 depletion significantly increased the production of photoreceptor cells and promoted photoreceptor-mediated ERG responses. In vitro, light specifically activated JNK1, but not JNK2. The activated JNK1 further phosphorylated the transcription factor c-Jun, which potentiated Notch1 transcription. The JNK1–c-Jun–Notch1 axis suppressed photoreceptor-related transcriptional factor expression and further impaired photoreceptor opsin expression (Figure 8).

Notch1 is known to determine the progenitor multipotency in a developing retina [45,46,47,48,49,50]. Notch signaling sustains the progenitor cells in an undifferentiated state. This action is mainly mediated by Notch1 downstream effectors, such as bHLH (Basic Helix-Loop-Helix) transcription factors [18,51,52]. The inhibition of Notch1 channeled the progenitors toward differentiating into rod or cone photoreceptor cells [20,21,22]. Our study filled a missing gap by demonstrating that light-activated JNK1 regulated Notch1 expression by phosphorylating the transcription factor c-Jun, which in turn bound to the cognate transcription regulatory region on Notch1.

Several gain- and loss-of-function studies of the JNK pathway have revealed that JNK3 has functions distinct from JNK1 or JNK2 due to its relative tissue specificity. The functions of JNK1 or JNK2 could be inferred from the differences in the phenotypes of *Jnk1* KO and *Jnk2* KO mice in terms of their contributions to the cellular regulation of the nervous system. Specifically, the phenotype of *Jnk1* KO mice is more dramatic than that of *Jnk2* KO mice, with abnormal brain development [53]. Furthermore, neurogenesis in vitro primarily requires JNK1, but not JNK2 or JNK3 [54]. Consistently, our finding also uncovered that JNK1, but not JNK2, regulated the production of photoreceptor cells.

While our study revealed that JNK1 activated Notch1 to negatively regulate photoreceptor gene expression, a previous study found that JNK1 could be a positive regulator for rhodopsin expression through interacting with and regulating Nrl transcriptional activity [55]. While the use of different cell culture models may have caused the result discrepancy, both studies revealed the crucial roles of JNK1 in regulating photoreceptor fate. Since both positive and negative signaling are commonly required for appropriate and smooth developmental transition and organ biogenesis, it is intriguing to speculate that JNK1 may play a dual role to fine-tune opsin expression during retinogenesis. Besides, it is also possible that JNK1 plays an opposite role in different developmental stages of retinogenesis. The role of JNK in retinogenesis and its detailed mechanisms still require further exploration.

The gene regulatory network of photoreceptor genesis is a research frontier for biomedical investigations. Aberrations in photoreceptors result in such retinal diseases as retinitis pigmentosa (RP) and age-related macular degeneration (AMD), which lead to visual impairment and blindness. Notch and Wnt signaling pathways have been proposed as potential targets to treat eye diseases, due to their fundamental importance in retinal development [56]. In our study, the JNK1–c-Jun–Notch1 axis influenced some transcriptional factors and three opsins, such as Crx and Rhodopsin, which are drug targets of retinitis pigmentosa [57]. It is possible that JNK1 becomes a potential target of eye diseases. It would be intriguing to explore the actions of the upstream regulators of JNK1 and integrate the network regulations of retinogenesis in future studies. The characterization of a new regulatory kinase or transcription factor will be instrumental for understanding the fine-tuning of photoreceptor genesis, ultimately providing novel therapeutic strategies to manipulate photoreceptor cells and improve vision accordingly.

## Supplementary Materials

The following are available online at https://www.mdpi.com/2073-4409/8/9/970/s1, Figure S1. Confirmation of Jnk1 and Jnk2 KO mice, Figure S2. The markers expression in Jnk1 KO and wild-type mice, Figure S3. The expression changes of *Jra* after 3 h blue light exposure in fly photoreceptor, Figure S4. The predicted c-Jun binding sites in the c-Jun ChIP-seq peak region.
