# Supplementary material for: JNK1 Induces Notch1 Expression to Regulate Genes Governing Photoreceptor Production
Source: Cells. 2019 Aug 24;8(9):970. doi: 10.3390/cells8090970 (PMC6769813; doi:10.3390/cells8090970)
Supplement: Supplementary file 1 [file cells-08-00970-s001.pdf]

cells
MDPI

Supplementary figures

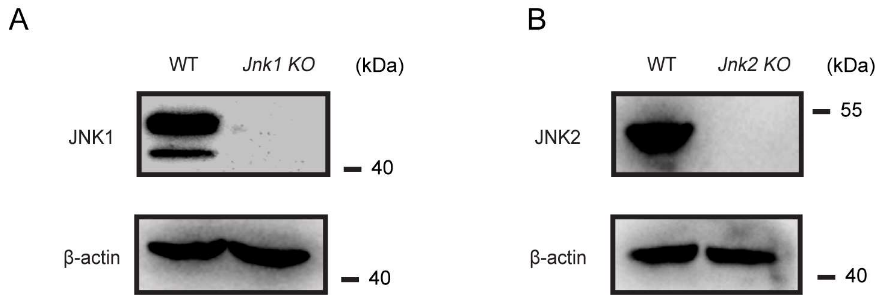

Figure S1. Confirmation of *Jnk1* and *Jnk2* KO mice. **A**, Eyes were isolated from WT and *Jnk1* KO mice at P30. Tissue homogenate was collected for western blot analysis using an anti-JNK1 antibody to confirm JNK1 ablation in the *Jnk1* KO retina. **B**, Eyes were isolated from WT and *Jnk2* KO mice at P30. Tissue homogenate was collected for western blot analysis using an anti-JNK2 antibody to confirm JNK2 ablation in the *Jnk2* KO retina.

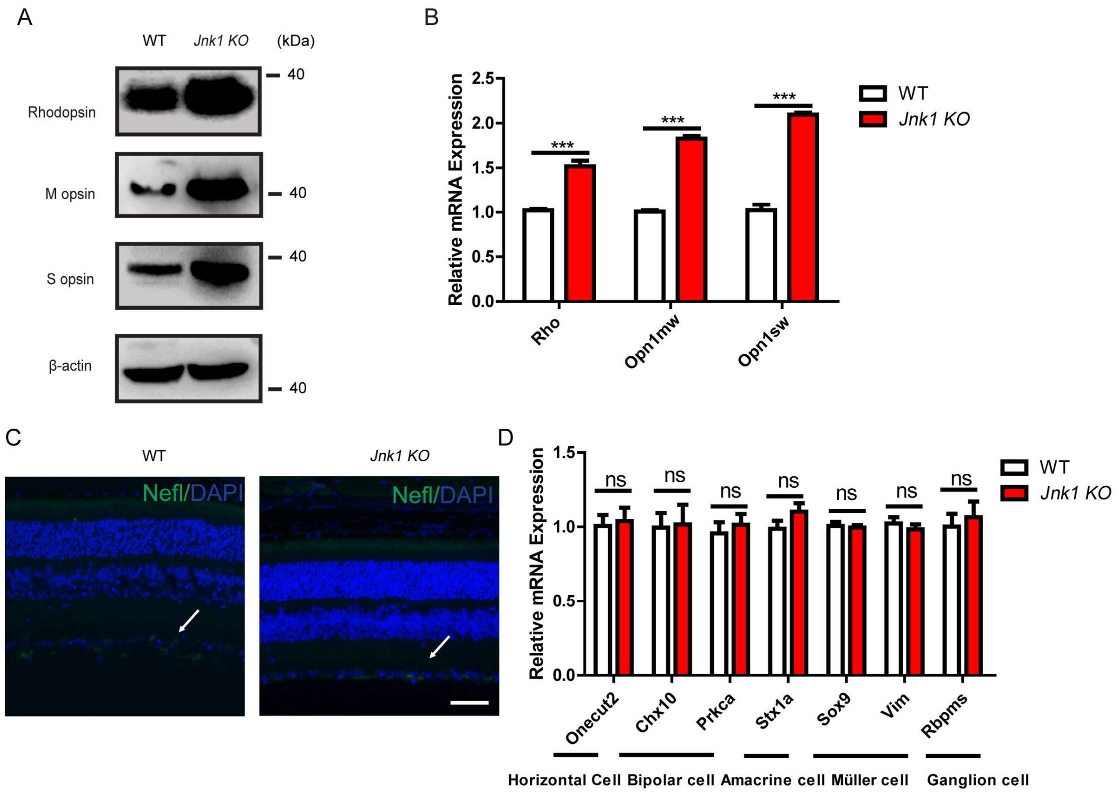

Figure S2. The markers expression in *Jnk1* KO mice. **A**, Eyes were isolated from WT and *Jnk1* KO mice at P30. Tissue homogenate was collected for western blot analysis using an anti-Rhodopsin antibody, anti-S-opsin antibody, anti-M-opsin antibody to indicate Rod cells, S cone cells, M cone cells respectively. **B**, Real-time PCR analysis of *Rho*, *Opn1sw*, *Opn1mw* mRNA in WT and *Jnk1* KO retinas at P30. Graphs show the mean ± SD, and the data shown are representative of three retinas. ***$p < 0.001$ (two-tailed Student's t-test). **C**, Section immunofluorescence on WT and *Jnk1* KO retinas at P30. Arrows pointed to the protein. Scale bars: 50 μm. **D**, Real-time PCR analysis of *Onecut2*, *Chx10*, *Prkca*, *Stx1a*, *Sox9*, *Vim*, *Rbpms* mRNA in WT and *Jnk1* KO retinas at P30. Graphs show the mean ± SEM, and the data shown are representative of three retinas. *** $p < 0.001$ (Wilcoxon-Mann-Whitney test).

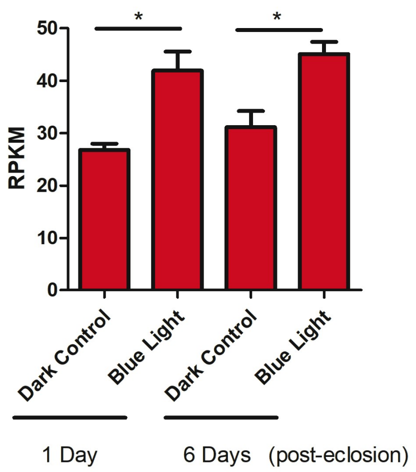

Figure S3. The expression changes of Jra after 3 h blue light exposure in fly photoreceptor**.** Jra, the orthologous gene of c-Jun of mouse, was significantly induced after 3 h blue light exposure in both 1 day and 6 days post-eclosion flies. The RNA-seq data were downloaded from GEO [32]. The gene expression quantification and differential expression were estimated using feature Counts and edgeR, respectively. Graph shows the mean ± SEM, and the data shown are representative of three replicates. * $p < 0.05$ (Negative binominal test, FDR < 5%).

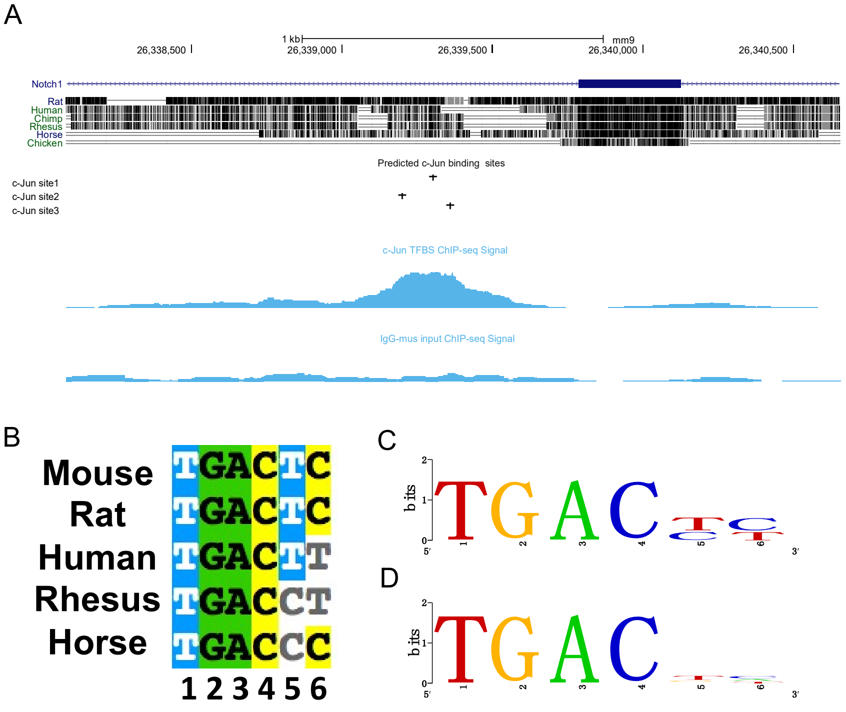

Figure S4. The predicted c-Jun binding sites in the c-Jun ChIP-seq peak region. (**A**), The five tracts show Notch1 gene structure, overall region sequence conservation of 7 vertebrate species, predicted c-Jun binding site, c-Jun ChIP-seq signal and IgG ChIP-seq signal. The c-Jun binding site was predicted using TRANSFAC based on the vertebrate non-redundant PWM (Position Weight Matrix) of c-Jun. (**B**), The multiple sequence alignment of c-Jun binding site 1 across five mammalian species (Mouse, Rat, Human, Rhesus and Horse). (**C**), The consensus sequence of c-Jun binding site 1 across five mammalian species represented using sequence logo. The consensus magnitude is measured using Shannon information content in bits. Low bit value represents all nucleotides occur with similar probability at specific position. (**D**), The consensus sequence of canonical c-Jun binding site based on PWM annotated by TRANSFAC (c-Jun, T00131).
